# Editorial-Special Issue “Macromolecular Self-Assembly Materials: From Modeling to Advanced Applications”

**DOI:** 10.3390/ma13061458

**Published:** 2020-03-23

**Authors:** Salvatore Magazù, Domenico Lombardo

**Affiliations:** 1 Dipartimento di Scienze Matematiche e Informatiche, Scienze Fisiche e Scienze della Terra, Università di Messina, 98166 Messina, Italy; 2 Consiglio Nazionale delle Ricerche, Istituto per i Processi Chimico-Fisici, 98158 Messina, Italy

Materials self-assembly represents a key strategy for the design and fabrication of nanostructured systems and has become a fundamental approach for the construction of advanced nanomaterials. The synthesis of novel chemical structures as well as the efficient use of soft and supramolecular interactions allow for the design of novel materials with nanoscale ordered morphologies, whereby the assemblies of different building blocks and their integrated actions facilitate the performance of highly specific functions suitable for novel and smart applications in material science and biotechnology. Moreover, the reversibility of the noncovalent forces allows a dynamic switching of the nanomaterials’ structural properties in response to various (internal/external) stimuli, and provide additional flexibility for the design and fabrication of smart nanomaterials and functional nano-platforms.

The Special Issue “*macromolecular self-assembly materials: from modeling to advanced application*” comprises one review article concerning the self-assembly processes involving organic materials [1], and three original research papers that provide important insights into the self-assembly process of inorganic [2] and hybrid materials [3,4]. Finally, the Special Issue included a theoretical study concerning the complex self-assembly process involved in a model system composed of functionalized hard–soft dimers [5].

The organic materials (building blocks) represent the best examples of biocompatible nanostructures for advanced biomaterial applications, as they present better characteristics and properties to match the physico-chemical condition of biological tissues. In the review article of Lombardo et al. [1], the authors analyze the main parameters that sensitively influence the design of organic nanostructured systems, while putting into evidence challenges, limitations, and emerging approaches in the various fields of nanotechology and biotechnology. The article analyzes the main features of the self-assembly of traditional amphiphiles (which represents the precursor of the bottom-up methods in modern nanoscience), which is characterized by the formation of various phases including micellar aggregates, vesicles, lamellar structures, nano- and microemulsions, and (lyotropic) liquid crystals nanostructures [1]. The article also analyzes the self-assembly processes of polymer nanomaterials obtained by the interaction of a variety of versatile polymer-based building blocks that include linear (block) copolymers, cross-linked polymers (nano-gels), and hyperbranched/dendritic polymers (such as dendrimers). Moreover, a variety of biomolecules including peptides, proteins (viruses and enzymes), lipids, and oligonucleotides (DNA/RNA) macromolecules exhibit great potential to form supramolecular functional nanostructures. Due to their excellent physico-chemical properties and their higher bio-reactivity, these biomolecules allow for the development of advanced nanodevices in the fields of materials science, biotechnology, and biomedical engineering. Finally, carbon nanotubes, graphene, and fullerene represent important classes of organic building blocks for the development of emerging nanotechnologies. Their self-assembly properties and main applications have been shortly reviewed in the final sections of the review article [1].

The Special Issue includes an interesting study of the layer self-assembly process promoted in montmorillonite–water mixtures during the dehydration process [2]. More specifically, in the investigation of Caccamo at al., experimental data, collected by Fourier transform infrared and Raman spectroscopies on montmorillonite–water mixtures at different concentration values and as a function of time, allowed for some insight to be obtained into the hydrogen bond network of water within the montmorillonite network. Two complementary approaches for the spectral data analysis are proposed in the paper: an analysis of the intramolecular OH stretching mode in terms of Gaussian components, and an analysis of the same OH stretching mode by an innovative wavelet cross-correlation approach. For both the FTIR and Raman spectra, the decomposition of the intramolecular OH stretching band into a “closed” and an “open” contribution and the spectral wavelet analysis allowed them to extract quantitative information on the time behavior of the system water content. It emerges that, while the total water contribution inside the montmorillonite structure decreases as a function of time, the relative weight of the ordered water contribution diminishes more rapidly with respect to the disordered water contribution. This result allows to infer that, during the dehydration process, the residual water content, characterized by a higher structural disorder, rests entrapped in the montmorillonite layer structure, promoting a layer self-assembly [2].

The Special Issue also includes two interesting studies concerning the self-assembly of hybrid nanomaterials [3,4]. Yu et al. [3] proposed a new facile microfluidic route (based on a three step assembly process) for the synthesis of novel core-shell composite/hybrid polymeric microparticles doped with organic and inorganic nanoparticles. The resulting microparticles, characterized by scanning electron microscopy and energy dispersive x-ray spectrometry, revealed the core-shell composite/hybrid polymeric morphology, the presence of silver nanoparticles in the shell, and the organic nanoparticles in the core, but failed to reveal the presence of the gold nanoparticles in the core, presumably due to their too small size (c.a. 2.5 nm). Nevertheless, the proposed three-step assembly approach allowed for the easy formation of composite/hybrid multi-scale and multi-domain polymeric microparticles suitable for a wide range of applications in the field of materials science.

In the article of Calogero at al. [4], five 2-styryl-1-benzopyrylium salts and their relative self-assembly processes toward TiO_2_ nanocrystalline layers (semiconductor substrate) were evaluated in their efficiency as photosensitizers in dye-sensitized solar cells (DSSCs). The spectroscopic and photoelectrochemical investigation conducted on these five bio-inspired dyes, in solution and upon adsorption onto titanium dioxide films, allowed for a detailed description of the anchoring ability as well as the ability as photosensitizers of the different donor groups decorating the 2-styryl-1-benzopyrylium core. The experimental results, which have been supported by theoretical calculations, evidenced that the introduction of a dimethylamino group in position 4′ of the styrylflavylium skeleton, can alter the conjugation of the molecule and facilitate a greater absorption in the visible region and a better injection electronic part of the dye to the conduction band of TiO_2_. The investigation may stimulate the design of novel targeted bio-inspired dye molecules, for the development of efficiency photosensitizers in dye-sensitized solar cells (DSSCs).

Finally, this Special Issue includes an interesting theoretical investigation concerning the self-assembly process of a dimeric model system characterized by well-defined interactions [5]. In their theoretical work, F. Sajia and G. Munaò investigated a hard–soft dimeric model system composed by two hard spheres belonging to different dimers that interact via a bare hard-core repulsion, whereas two soft spheres experience a softly repulsive Hertzian interaction [5]. By performing Monte Carlo (MC) simulations and integral equation calculations, they explored a wide range of temperatures and densities. More specifically, the fluid phase behavior, which was investigated by analyzing the structural and thermodynamic properties of the system (including radial distribution functions, structure factors, pair correlation entropy, average cluster size and bonds distribution), allowed them to identify the existence of specific structural inhomogeneities that indicate the possible onset of aggregate formation, even if no attraction is present in the investigated system. The proposed model may serve as a useful framework for a more systematic investigation of self-assembled nanostructures of functionalized hard–soft dimers as well as for more complex systems that are able to undergo self-assembly processes in the field of colloids and complex macromolecular systems.

Overall, the collection of the articles published in this Special Issue may help to identify the fundamental factors involved in the self-assembly of nanostructures by analyzing the main parameters that sensitively influence the design of nanostructured systems, while putting into evidence challenges, limitations, and emerging approaches in the various fields of nanotechology and biotechnology. Finally, the Guest Editors would like to sincerely thank all the authors for their valuable contributions.
